# Synergistic Effects of Lignin Type and Polyethylene Glycol on the Structure Property Relationships of PLA/Lignin Biocomposites

**DOI:** 10.3390/polym18192353

**Published:** 2026-09-27

**Authors:** Monpilai Narasingha, Shiva Lall Sunar, Suchata Kirdponpattara, Malinee Sriariyanun, Elizabeth Jayex Panakkal, Santi Chuetor

**Affiliations:** 1 Department of Chemical Engineering, Faculty of Engineering, King Mongkut’s University of Technology North Bangkok (KMUTNB), Bangkok 10800, Thailand; monpilai.n@eng.kmutnb.ac.th (M.N.); suchata.k@eng.kmutnb.ac.th (S.K.); 2 Department of Chemical and Biological Engineering, Montana State University, Bozeman, MT 59717, USA; shivalall.sunar@montana.edu; 3 Chemical and Process Engineering, The Sirindhorn International Thai German International Graduate School of Engineering, King Mongkut’s University of Technology North Bangkok (KMUTNB), Bangkok 10800, Thailand; macintous@gmail.com (M.S.); elizabeth.jayex@gmail.com (E.J.P.); 4 Centre of Eco-Materials and Cleaner Technology, King Mongkut’s University of Technology North Bangkok (KMUTNB), Bangkok 10800, Thailand

**Keywords:** bioplastics, lignin reinforcement, polylactic acid, sodium lignosulfonate, electric vehicles, melt flow rate, thermal stability, sustainable composites

## Abstract

The growing electric vehicle (EV) industry necessitates sustainable material solutions to reduce reliance on petroleum-based plastics. This study investigates lignin-reinforced polylactic acid (PLA) biocomposites for sustainable EV components, focusing on rearview mirror covers. Alkaline lignin (AL) and sodium lignosulfonate (LS) were incorporated into PLA at 0–3 wt.% with polyethylene glycol (PEG400) as a compatibilizer. The objective was to develop a bio-based composite suitable for non-structural EV components, specifically a rearview mirror cover. The composites were characterized via scanning electron microscopy (SEM), universal testing machine (UTM), thermogravimetric analysis (TGA), and melt flow rate (MFR) testing. Results revealed that LS-PLA composites exhibited superior mechanical strength (38.6 MPa tensile strength at 1 wt.%) and thermal stability (onset degradation: 347.9 °C) compared to AL-PLA, attributed to enhanced interfacial compatibility. PEG increased MFR by 40–60% but reduced tensile strength by 15–20% due to plasticization. Optimal performance was achieved with 1 wt.% LS, demonstrating balanced processability (MFR: 12 g/10 min), thermal resilience, and economic viability (IRR: 28.5%). These biocomposites reduce petroleum dependency while meeting EV industry standards, supporting circular economy goals.

## 1. Introduction

The escalating climate crisis and stringent environmental regulations are compelling the automotive industry to transition towards sustainable manufacturing practices. This shift is particularly critical for the electric vehicle (EV) sector, where the environmental benefits of zero tailpipe emissions can be undermined by the significant carbon footprint associated with vehicle production, especially from conventional petroleum-based materials. Consequently, there is a pressing demand for advanced biocomposites that offer enhanced performance characteristics while aligning with principles of circular economy and reduced environmental impact [1]. The scale of the challenge is underscored by global plastic production, which reached nearly 335 million tons in 2021 and is projected to soar to approximately 600 million tons by 2030, without intervention [2]. This reliance on fossil-fuel-based plastics is unsustainable, highlighting an urgent need for renewable alternatives.

The pursuit of sustainability in the EV industry must be balanced with the uncompromising demand for performance. Plastics thus play a paradoxically critical role here. Lightweighting is a fundamental engineering strategy for extending the limited range of EV batteries, and high-performance plastics are indispensable for replacing heavier metal components without sacrificing structural integrity. This importance is vividly reflected in market projections. The dedicated EV plastics market is estimated to be valued at USD 3.92 billion in 2025 and is expected to expand at a remarkable compound annual growth rate (CAGR) of 28.13% to reach USD 13.54 billion by 2030 [3]. This exponential growth underscores a pivotal opportunity, i.e., to replace conventional plastics with bio-based alternatives at the inception of this market expansion, thereby embedding sustainability into the fabric of the evolving EV ecosystem.

Among bio-based polymers, polylactic acid (PLA) has emerged as a front-runner due to its biodegradability, renewability, and relatively well-established production processes. However, the direct substitution of conventional engineering plastics in demanding automotive applications with neat PLA is hampered by several intrinsic limitations. Its inherent brittleness, low heat resistance (low glass transition temperature, *T_g_*), and slow crystallization rate limit its broader applications. Its susceptibility to moisture absorption further restricts its use in components exposed to mechanical stress, thermal cycling, and varying environmental conditions [4,5]. Therefore, for PLA to be a viable material for EV components (ranging from interior trim to under-the-hood parts), its mechanical robustness and thermal stability must be significantly enhanced.

A promising strategy to overcome PLA’s weaknesses is through reinforcement with natural fillers. The use of natural fibers as reinforcement in biocomposites has garnered significant research interest due to their eco-friendly properties and potential to reduce reliance on synthetic materials [6]. Among these, lignin, a complex polyphenolic polymer and a major constituent of plant cell walls, presents a uniquely attractive opportunity. It is an abundant byproduct of the pulp and paper industry, with emerging lignocellulose biorefineries contributing to a vast supply; approximately 100 million tons were generated in 2015 alone [7]. Despite an industrial lignin market value projected to rise to USD 913 million by 2025, primarily sourced from agricultural and timber waste. The majority of this valuable bioresource is currently underutilized for low-value energy generation. This represents a significant waste of a potential bioresource. The incorporation of lignin as a reinforcing filler in PLA composites not only valorizes this waste stream but also offers a sustainable pathway to reduce the environmental footprint of the resulting materials [8]. Furthermore, natural fiber-reinforced biopolymers, a category including lignin-–PLA composites, have demonstrated notable improvements in tensile and flexural strengths, attributed to strong interfacial adhesion between the fiber and the polymer matrix [9].

Despite its promise, the integration of lignin into PLA faces a significant scientific hurdle, i.e., compatibility. Lignin’s heterogeneous and highly polar chemical structure often leads to poor dispersion within the relatively hydrophobic PLA matrix, resulting in agglomeration and weak interfacial adhesion [10]. This incompatibility can severely compromise the mechanical properties it is intended to improve [11,12]. To address this, researchers have begun exploring specific lignin types with more favorable chemical properties. Recent studies suggest that sodium lignosulfonate (LS), a lignin derivative containing hydrophilic sulfonate groups, may offer superior compatibility with certain polymers. These sulfonate groups can potentially enhance interfacial adhesion with PLA, leading to a more homogeneous composite and better stress transfer [13]. A comparative study between standard alkali lignin (AL) and LS within a PLA matrix is therefore critical to understanding how lignin chemistry influences composite performance.

A common strategy to mitigate dispersion issues in polymer composites is the use of compatibilizers or plasticizers. Polyethylene glycol (PEG) has been frequently employed in PLA-based systems for this purpose. PEG can intercalate between polymer chains, increasing free volume and chain mobility, which enhances the processability and ductility of the inherently brittle PLA [14,15]. This plasticizing effect can aid in the dispersion of lignin particles. However, this advantage involves a trade-off. The plasticizing effect that enhances ductility can reduce tensile strength and modulus, which may limit the mechanical properties of PLA-based composites [16,17]. The central hypothesis of this work is that PEG may play a more synergistic role when used with specific lignin types. Rather than simply plasticizing the PLA matrix, PEG could act as a molecular bridge, improving the interfacial adhesion between lignin and PLA by interacting with the polar groups on both components. This could potentially unlock enhanced properties that are not achievable by either additive alone.

While previous studies have independently explored lignin reinforcement or PEG plasticization of PLA, a comprehensive investigation into their synergistic effects is lacking. Prior works have not sufficiently addressed the interplay between different lignin chemistries (e.g., AL vs. LS) and a plasticizer like PEG under the combined thermal and mechanical stresses typical of automotive environments. Moreover, few studies integrate a rigorous techno-economic analysis with material optimization, which is essential for assessing the real-world viability of these biocomposites for industrial applications like EV component manufacturing [18,19]. This points towards a critical research gap. The combination of lignin type and PEG content can be optimized to create a biocomposite that meets the performance metrics for EV applications while remaining economically feasible.

This research therefore aims to systematically investigate the synergistic effects of various lignin types and PEG on the mechanical, thermal, and rheological properties of PLA biocomposites, with the ultimate goal of optimizing their performance for demanding EV applications. The integration of lignin, a readily available and cost-effective biopolymer, with polylactic acid presents a promising avenue for developing environmentally benign alternatives to conventional petroleum-based plastics [20,21]. This approach directly addresses the limitations of both pristine PLA and traditional composites, paving the way for sustainable materials suitable for diverse automotive applications [22,23]. 

The specific objectives of this study are to as follows: i. To to compare the reinforcement efficacy of AL and LS at low loading levels (0–3 wt.%) within a PLA matrix; ii. To quantify the role of PEG400 (fixed at 5 wt.%) in modifying the compatibility, morphology, and performance of the AL/PLA and LS/PLA composites; iii. To to comprehensively evaluate the resulting biocomposites based on key performance metrics for automotive applications, including mechanical (tensile, flexural, impact), thermal (TGA), and rheological properties; iv. To to conduct a preliminary techno-economic analysis (TEA) to assess the feasibility of utilizing the optimized biocomposite for the production of a specific EV component, such as a rearview mirror housing, benchmarking it against conventional materials. By integrating advanced material characterization with economic assessment, this study establishes a comprehensive framework for developing high-performance and sustainable biocomposites.

## 2. Materials and Methods

### 2.1. Materials

PLA, grade Luminy^®^ LX175 (Poly (D, L-lactic acid)), with a weight-average molecular weight (Mw) of approximately 115,000 g/mol, was supplied by Nature Works LLC (Minnetonka, MN, USA). Two types of lignin were employed as reinforcing fillers: alkali lignin (AL), product code L0082, was procured from Sigma-Aldrich (St. Louis, MO, USA) with particle size distribution of ~100–250 μm, and sodium lignosulfonate (LS), product code L0098, with particle size distribution of ~250–400 μm was obtained from Tokyo Chemical Industry (TCI, Tokyo, Japan). PEG with an average molecular weight of 400 g/mol (PEG400), functioning as a plasticizer and potential compatibilizer, was supplied by Merck KGaA (Darmstadt, Germany). All materials were used as received, unless otherwise specified.

### 2.2. Composite Fabrication

#### 2.2.1. Material Drying

Due to the hygroscopic nature of both PLA and lignin, all raw materials were subjected to drying prior to processing in order to minimize hydrolytic degradation. PLA pellets were dried in a vacuum oven (Model DZF-6050, MTI Corporation, Richmond, CA, USA) at 80 °C for 24 h. Lignin powders (AL and LS) were similarly dried at 70 °C for 24 h to remove residual moisture.

#### 2.2.2. Melt Blending and Compounding

The biocomposites were prepared via melt blending using a co-rotating twin-screw extruder (Model PSHJ-20, XINDA Machinery, Quanzhou, China) with a screw diameter of 20 mm and an L/D ratio of 40. The formulations of the prepared composites are detailed in Table 1. Neat PLA was also processed under identical conditions to serve as a baseline control. PEG400 was incorporated at a fixed concentration of 5 wt.% in the PEG-containing formulations. This loading was selected to investigate its effect as a plasticizer and potential compatibilizer while maintaining a practical balance between improved processability and mechanical performance. Higher and lower PEG concentrations were not investigated in the present study.

The pre-weighed components were manually dry-blended to ensure a homogeneous initial mixture before feeding into the extruder. The feeding and compression temperature was set at 130 °C. The operating temperature was fixed at 130 °C since the glass transition temperature (T_g_) was typically from 50 to 60 °C. The screw speed was maintained at 150 rpm. The extruded strands were water-cooled and subsequently pelletized.

#### 2.2.3. Compression Molding

The extruded pellets were dried again at 80 °C for 6 h before being processed into standard test specimens using a compression molding machine (Model QC-601T, Cometech Testing Machines, Taichung, Taiwan). The pellets were placed in a pre-heated mold and subjected to a pre-load for 2 min at 180 °C, followed by a full pressure of 2000 kg/cm^2^ for 4 min. The mold was then transferred to a cold press and cooled to room temperature (24 °C) under pressure to produce sheets of appropriate thickness for mechanical and morphological characterization [24].

### 2.3. Mass Balance Analysis

A systematic mass balance was established to translate the laboratory-scale formulations into a consistent basis for the TEA. The balance was performed for the optimal composite formulation (PLA/LS1%) at a conceptual production scale of 100 kg of composite per day, operating for 300 days per year.

The process was modeled as a steady-state operation with no chemical reactions, implying that mass is conserved through each unit operation (i.e., Input = Output). The calculations were based on the weight percentages outlined in Table 1. The flow of materials through the primary processing steps- drying, extrusion, and compression molding is summarized in a block flow diagram.

The overall mass balance for the PLA/LS1% composite is as follows:

- Total Input: PLA: 99 kg; LS: 1 kg
- Total Output:100 kg
- Total Output: PLA/LS1% Composite Pellet/Product: 100 kg

This 1:1 mass conversion ratio confirms that the composition by weight is directly transferred from the raw materials to the final product, with no expected mass losses during processing under ideal conditions. This balance provided the foundational data for calculating the annual raw material requirements and associated costs presented in the techno-economic analysis.

### 2.4. Characterization Techniques

#### 2.4.1. Scanning Electron Microscopy (SEM)

The morphological properties and fracture surfaces of the biocomposites were examined using a Field Emission Scanning Electron Microscope (FEI QUANTA 450, Thermo Fisher Scientific, Waltham, MA, USA). The samples were cryo-fractured after immersion in liquid nitrogen to obtain a clean brittle fracture surface. The fractured surfaces were sputter-coated with a thin layer of gold to prevent charging and were imaged at accelerating voltages of 5–10 kV under various magnifications (1000× to 5000×) to assess lignin dispersion, interfacial adhesion, and overall composite morphology [25].

#### 2.4.2. Mechanical Testing

Tensile properties were determined according to the ASTM D638 standard [26] (Type V specimens) using a Universal Testing Machine (Instron 5567, Norwood, MA, USA) equipped with a 5 kN load cell. A minimum of five (5) specimens for each composition were tested at a crosshead speed of 5 mm/min at room temperature (24 °C). The values for tensile strength, Young’s modulus, and elongation at break were recorded and averaged [27].

#### 2.4.3. Thermogravimetric Analysis (TGA)

The thermal stability of the samples was evaluated using a Thermogravimetric Analyzer (TGA/DSC 3+, METTLER TOLEDO, Greifensee, Switzerland). Samples weighing approximately 5–10 mg were placed in alumina crucibles and heated from 25 °C to 900 °C at a constant rate of 10 °C/min under a continuous nitrogen purge flow of 50 mL/min. The onset decomposition temperature (Tonset, taken at 5% weight loss) and the temperature of maximum degradation rate (Tmax) were determined from the resulting thermograms [28].

#### 2.4.4. Melt Flow Rate (MFR)

The MFR, an indicator of melt processability and molecular weight, was measured using a Melt Flow Indexer (KARGE MeltFloW, Krailling, Germany) according to ISO 1133-1:2022 [29]. The test was conducted at 190 °C with a standard load of 2.16 kg. The MFR value, reported in g/10 min, was calculated as the average of three measurements for each sample. Figure 1 below illustrates the step-wise experimental procedure followed during the study.

### 2.5. Techno-Economic Analysis (TEA)

A preliminary TEA was conducted to evaluate the financial viability of producing an automotive component (specifically, a rearview mirror housing) from the optimized PLA/lignin biocomposite. The analysis was based on a production scale of 50,000 units per year, operating for 300 days annually. The analysis considered major cost factors, including i. Capital Investment: Costs for primary equipment (extruder, compression molding machine), auxiliary systems, and installation ii. Operating Costs: Raw material consumption (based on formulations in Table 1), utilities (electricity, water), and direct labor costs iii. Product Pricing: The selling price of the mirror housing was benchmarked against equivalent components made from conventional polymers like ABS.

Key financial metrics were calculated over a 10-year project horizon- i. Net Present Value (NPV): To determine the project’s profitability in present-value terms, using a discount rate of 10% ii. Internal Rate of Return (IRR): To calculate the projected percentage return on investment iii. Payback Period: To estimate the time required to recoup the initial capital investment from net cash flows. This model provided a first-order assessment of the economic feasibility and potential competitiveness of the developed biocomposite in an industrial setting.

## 3. Results

### 3.1. Morphological Analysis: The Role of Lignin Type and Polyethylene Glycol on Composite Structure

The biomass-derived materials have attreacted considerable interest as sustainable alternatives to conventional polymer additives [30]. Materials like lignin can be extracted via different techniques [31,32,33], and their incorporation into PLA composites can improve material sustainability while utilizing renewable resources. The surface morphology of a polymer composite plays an important role in determining the distribution of the reinforcement phase within the polymer matrix and the interactions between the two phases at their interface. The dispersion of the filler within the polymer matrix and the quality of the interfacial adhesion are critical determinants of composite performance. SEM analysis of the cryo-fractured surfaces provided clear evidence of the impact of lignin type and the presence of PEG. Figure 2 presents representative SEM micrographs of PLA/LS1, PLA/AL1, and PLA/PEG5/LS1 composites. These representative SEM micrographs were used to qualitatively compare the effect of lignin type and PEG incorporation on the composite morphology. The SEM images provide qualitative information on the dispersion and interfacial morphology of the lignin-containing PLA composites. As shown in Figure 2a, composites with 1 wt.% LS (PLA/LS1) exhibited a relatively homogeneous morphology with well-dispersed LS particles and no significant voids or pull-outs. This suggests good interfacial adhesion between the LS and the PLA matrix [34,35]. In contrast, composites with 1 wt.% AL (PLA/AL1) showed visible signs of agglomeration and poorer wetting by the matrix, leading to micro-voids around the AL particles. These morphological features suggest comparatively less uniform dispersion and weaker interaction between AL and the PLA matrix than observed for LS. The incorporation of 5 wt.% PEG400 significantly altered the composite morphology. The micrographs of the PLA/PEG5/LS1 composite (Figure 2c,d) showed a noticeable reduction in surface roughness compared to the PEG-free counterpart [36]. The lignin particles appeared to be more seamlessly integrated into the matrix, suggesting that PEG acted as a compatibilizing agent by improving polymer chain mobility and wetting the lignin particles, thereby reducing interfacial tension [37,38].

### 3.2. Mechanical Properties: Balancing Strength, Stiffness, and Ductility

The tensile properties of the biocomposites, summarized in Figure 3, directly reflect the morphological observations. The type and content of lignin, as well as the addition of PEG, had a profound impact on their mechanical behavior. Table 2 summarized the mechanical properties including tensile stress, strain and elongation at break of different biocomposites. The tensile strength and Young’s modulus were maximized at a low lignin loading of 1 wt.%. The PLA/LS1 composite achieved a tensile strength of 38.6 MPa and a modulus of 2.1 GPa, representing a significant improvement over neat PLA and a 61% higher strength than the PLA/AL1 composite (23.9 MPa) [39]. This enhancement is directly attributable to the superior dispersion and interfacial adhesion of LS, as seen in the SEM analysis, which allows for effective stress transfer from the polymer matrix to the reinforcing lignin particles [40,41]. However, at 3 wt.% loading, the tensile strength declined by approximately 40% for both lignin types due to filler aggregation acting as stress concentration points [42,43].

The addition of PEG400 introduced a standard plasticization effect [44]. While it decreased the elongation at break, enhancing the inherent brittleness of PLA, it reduced the tensile strength of neat PLA. PLA exhibited the highest elongation at break (2.8%), indicating the greatest tensile deformation among the tested samples while the incorporation of lignin (LS) or alkaline lignin (AL) generally reduced elongation at break compared with neat PLA. The relatively low elongation values across all samples indicate that these materials exhibit limited ductility and predominantly rigid mechanical behavior under the tested conditions. In contrast, Young’s modulus showed a slight increase from approximately 1.85 GPa to 1.99 GPa after the addition of 5 wt.% PEG400. A similar reduction in tensile strength was observed in the lignin-containing formulations after PEG400 incorporation, indicating that the effect of PEG400 on tensile strength remained evident in the presence of lignin. Thus, the combined use of lignin and PEG400 involves a trade-off between improved ductility and processability and reduced tensile strength and stiffness.

### 3.3. Rheological Properties and Melt Processability

The MFR, an indicator of melt viscosity and processability, is presented in Figure 4. In general, the incorporation of lignin increased the MFR, suggesting a reduction in melt viscosity. This is likely due to a chain-scission effect where lignin acts as a chain-terminating agent during processing, effectively reducing the average molecular weight of the polymer [45,46]. Composites with AL exhibited a higher MFR (peaking at 45.2 g/10 min for PLA/AL3) than those with LS (41.1 g/10 min for PLA/LS3). This difference reinforces the mechanical data, indicating stronger interactions between LS and the PLA chains, which restrict flow more effectively than the poorly adhering AL particles.

On the other hand, the plasticizing effect of PEG was dominant in the rheological properties. The incorporation of 5 wt.% PEG400 increased the MFR by 40–60% across all compositions. For instance, the MFR of PLA/PEG5/LS3 was 60.1 g/10 min which indicated that the PEG decreased the molecular interaction between PLA and enahnced its flowability of biocomposites. This substantial increase signifies a major improvement in flow characteristics, which is highly beneficial for injection molding or extrusion of complex-shaped components like EV mirror housings, as it can lead to lower processing temperatures and shorter cycle times [47,48].

### 3.4. Thermal Stability for Automotive Environments

The thermal degradation behavior, assessed by TGA, is crucial for evaluating the suitability of these biocomposite materials for automotive applications where components may be exposed to elevated temperatures. The key parameters are summarized in Table 3. LS-based composites demonstrated superior thermal resilience compared to AL-based ones [49]. The TGA graph showed that it was a single-stage degradation which indicated the degradation of main polymer composite (PLA). The onset degradation temperature (T_onset_ at 5% weight loss) for PLA/LS1 was 327.5 °C, which was 32.3 °C higher than that of PLA/AL1 (295.2 °C) and only slightly lower than neat PLA (342.2 °C) as seen in Figure 5. This can be attributed to the more stable aromatic structure and the char-forming tendency of LS, which acts as a thermal barrier [50,51]. This is further supported by the higher char residue retained by LS composites (12–15%) compared to AL composites (8–10%).

As expected, the addition of PEG400 reduced the thermal stability, lowering the onset temperature by 40–50 °C compared to its neat PLA which was due to the earlier degradation of the PEG component [52,53]. However, it is critical to note that even the composite with the lowest onset temperature (PLA/PEG5/LS1 at 288.5 °C) far exceeds the typical maximum cabin temperature of an EV (approximately 67 °C), confirming their thermal adequacy for interior applications.

### 3.5. Implications of Mass Balance on Process Scalability and Economic Feasibility

The mass balance analysis performed for the PLA/LS1% composite is a critical step in validating the scalability and economic potential of this biocomposite (Figure 6). The confirmation of a closed mass balance (Input = Output) under assumed ideal conditions underscores a key advantage of melt-blending processes- high material utilization efficiency.

This efficiency directly contributes to the promising economic viability indicated by the TEA (IRR of 28.51%). The direct proportionality between raw material input and product output simplifies cost forecasting and reduces uncertainty in scale-up. Any minor deviations from this ideal balance in an industrial setting, such as material loss due to purging or trimmings, can be incorporated as a known scaling factor, making the financial model robust.

Furthermore, the mass balance directly links the enhanced material properties of the PLA/LS1% composite to its economic value. The superior mechanical and thermal properties of this formulation, attributed to the good compatibility of LS with the PLA matrix, are achieved without complex chemical modifications or costly purification steps. The simple physical mixing process, accurately represented by the mass balance, results in a high-value product from relatively low-cost and sustainable inputs (PLA and lignin). The mass balance demonstrates the compatibility of high material performance with a straightforward processing route. These observations suggest that the developed biocomposite has strong potential for sustainable EV components from both technical and commercial perspectives.

In summary, the mass balance highlights the potential of the optimal PLA/LS1% composite as a scalable material solution from laboratory development to industrial manufacturing. The combination of efficient material utilization, enhanced performance, and economic feasibility supports its suitability for future large-scale applications.

### 3.6. Techno-Economic Viability for EV Component Manufacturing

A preliminary TEA was conducted to assess the feasibility of manufacturing a rearview mirror housing from the optimized PLA/LS1 composite at a scale of 50,000 units per year. The analysis, summarized in Table 4, showed promising results.

The production cost was estimated at $0.72 per unit. The projected financial metrics over a 10-year horizon indicate strong commercial potential, with an IRR of 28.5% and a NPV of $18,160. A sensitivity analysis revealed that the operation remains profitable even if the selling price drops to $3.90 per unit, providing a significant buffer against market fluctuations. The attractive IRR and a calculated payback period of less than 4 years demonstrate the economic viability of scaling up this sustainable material for automotive applications. It should be noted that the present TEA represents a preliminary economic assessment based on the assumed production conditions and manufacturing configuration. The effects of material losses, manufacturing scrap, and processing overheads may influence the actual production cost and economic performance. Furthermore, although compression molding was considered in this preliminary assessment, injection molding may offer greater suitability for the high-volume manufacturing of complex automotive components. A comprehensive comparison of alternative manufacturing technologies, incorporating realistic material yields and process-specific operating costs, is therefore recommended for future industrial-scale techno-economic evaluation.

## 4. Discussion

### 4.1. Synergistic Interplay Between Lignin Type and Polyethylene Glycol

The results clearly demonstrate that the type of lignin is a critical factor governing the properties of PLA biocomposites [54,55]. The superior performance of LS over AL, both mechanically and thermally, is primarily due to the presence of sulfonate groups [56]. These polar groups facilitate stronger interfacial adhesion with PLA via polar interactions and potential hydrogen bonding, leading to better dispersion, as confirmed by SEM, and more efficient stress transfer, resulting in higher tensile strength and modulus. According to a study, adding 6% of either lignosulfonate or AL enhanced the yield tensile strength of protein films isolated from soy by 63% and 114%, respectively [57].

PEG400 plays a dual role. Its primary function is as a plasticizer, increasing chain mobility, which manifests as higher elongation at break and a significantly increased MFR. The addition of PEG400 reduced the tensile strength, and a similar reduction in tensile strength was observed in lignin-containing composites, indicating that the effect of PEG400 on tensile strength remained evident in the presence of lignin. For neat PLA, however, the Young’s modulus showed a slight increase after the addition of 5 wt.% PEG400. Since no additional characterization was performed to determine the specific cause of this change, the observed increase in Young’s modulus is reported as an experimental result without assigning a definitive mechanism. Thus, the tensile-strength reduction following PEG400 incorporation was also observed in the lignin-containing composites, which indicates plasticizing effect of PEG400 remained significant even in the presence of lignin. Therefore, the reinforcing effect of lignin and the plasticizing effect of PEG400 should be considered together when optimizing the composite formulation. Interestingly, the morphological analysis suggests a secondary, beneficial role as a processing aid that improves the wetting and dispersion of lignin, particularly LS. The trade-off between enhanced processability/ductility and changes in strength and stiffness must be carefully balanced based on the specific application requirements.

### 4.2. Implications for Electric Vehicle Applications

The optimized PLA/LS1 composite meets or exceeds several key requirements for non-structural EV interior components such as rearview mirror housings, interior trim, and storage compartments.

- Mechanical Performance: With a tensile strength of 38.6 MPa and a modulus of 2.1 GPa, it is competitive with conventional plastics like ABS (typically ~40 MPa tensile strength) used for such parts.
- Thermal Stability: Its onset degradation temperature above 300 °C provides a substantial safety margin against the thermal loads encountered inside an EV cabin.
- Processability: The favorable MFR, especially when PEG is added, indicates excellent suitability for high-volume manufacturing processes like injection moulding.
- Sustainability: Most importantly, this composite provides a partially bio-derived alternative to conventional petroleum-based plastics. Its application in automotive components could support decarbonization efforts by potentially reducing CO_2_ emissions by up to 60%.

### 4.3. Economic and Environmental Impact

The techno-economic analysis confirms that the proposed biocomposite is not just a laboratory curiosity but an economically viable material. At an IRR of 28.5%, the project presents an attractive investment opportunity. Scaling this technology to a broader market penetration, for instance, supplying components for 5% of the ASEAN EV market (estimated at 160,000 units/year), could significantly valorize lignin waste streams from the pulp and paper industry. This approach directly supports United Nations Sustainable Development Goals (SDGs) 9 (Industry, Innovation, and Infrastructure) and 12 (Responsible Consumption and Production), creating a circular economy model for the automotive sector.

## 5. Conclusions

This study successfully demonstrates the optimization of lignin-reinforced PLA biocomposites for electric vehicle applications. The key findings are:

- LS is a more effective reinforcement for PLA than AL due to superior interfacial adhesion, leading to enhanced tensile strength (38.6 MPa at 1 wt.%) and thermal stability (onset degradation at 347.9 °C).
- PEG400 acts as an effective plasticizer and processing aid, significantly improving melt flow (40–60% increase in MFR) and ductility, albeit with a trade-off in tensile strength.
- The PLA/LS1 composite formulation exhibits an optimal balance of mechanical, thermal, and rheological properties that meet the requirements for EV interior components.
- A comprehensive TEA confirms the financial viability of producing a rearview mirror housing from this biocomposite, with an attractive IRR of 28.5% and a short payback period.

Future studies will explore surface-modified lignin nanoparticles for further improvement of composite properties. Additional efforts will focus on lifecycle assessment and long-term durability testing under simulated automotive conditions to evaluate environmental impacts and practical performance.

## Figures and Tables

**Figure 1:**
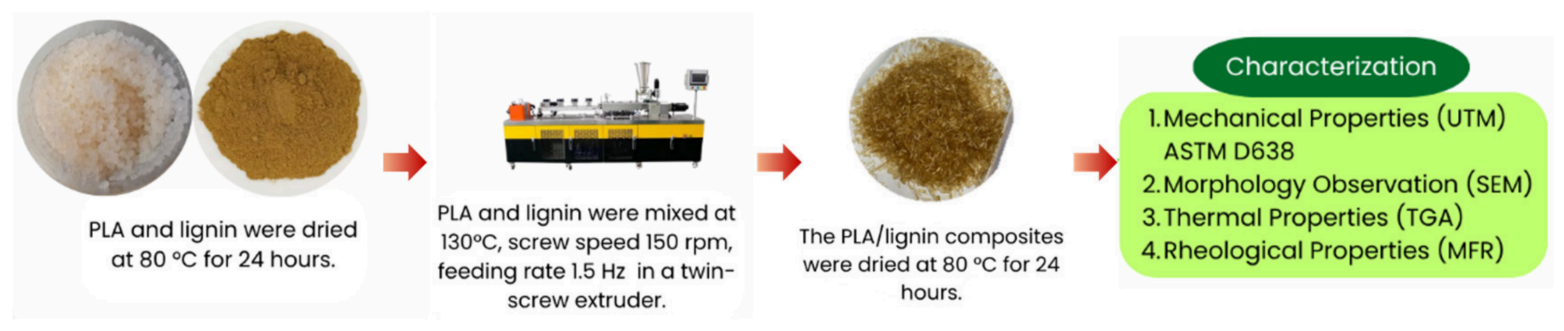
Experimental procedure of the study.

**Figure 2:**
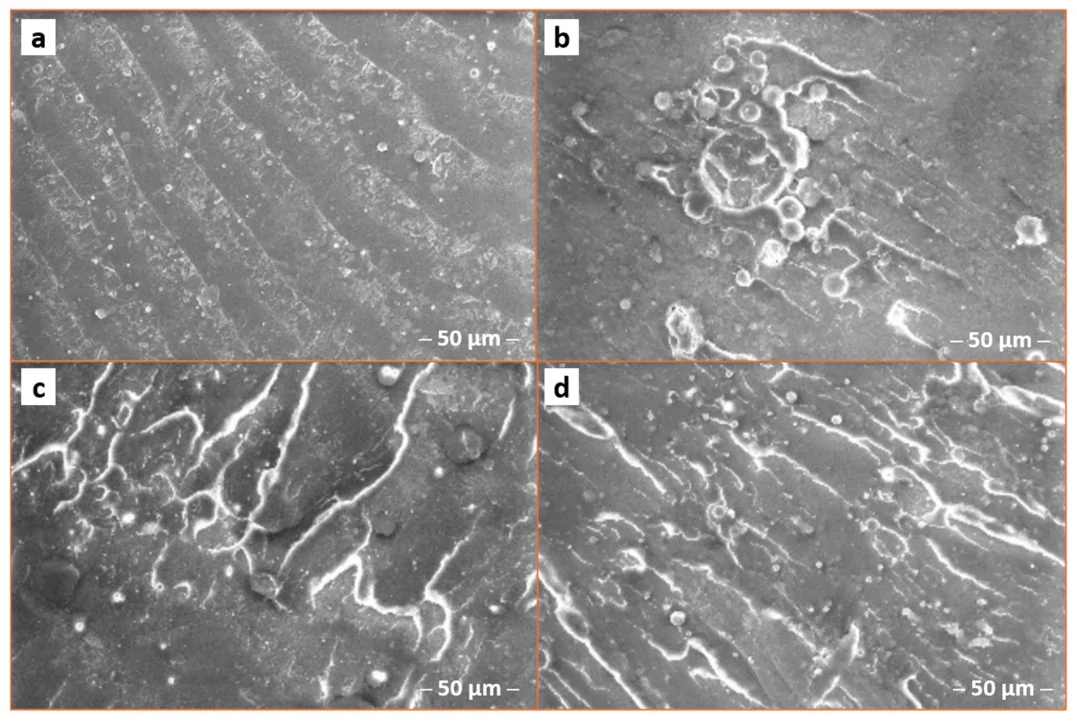
SEM micrographs of PLA biocomposites: (**a**) PLA/LS1; (**b**) PLA/AL1; and (**c**,**d**) PLA/PEG5/LS1.

**Figure 3:**
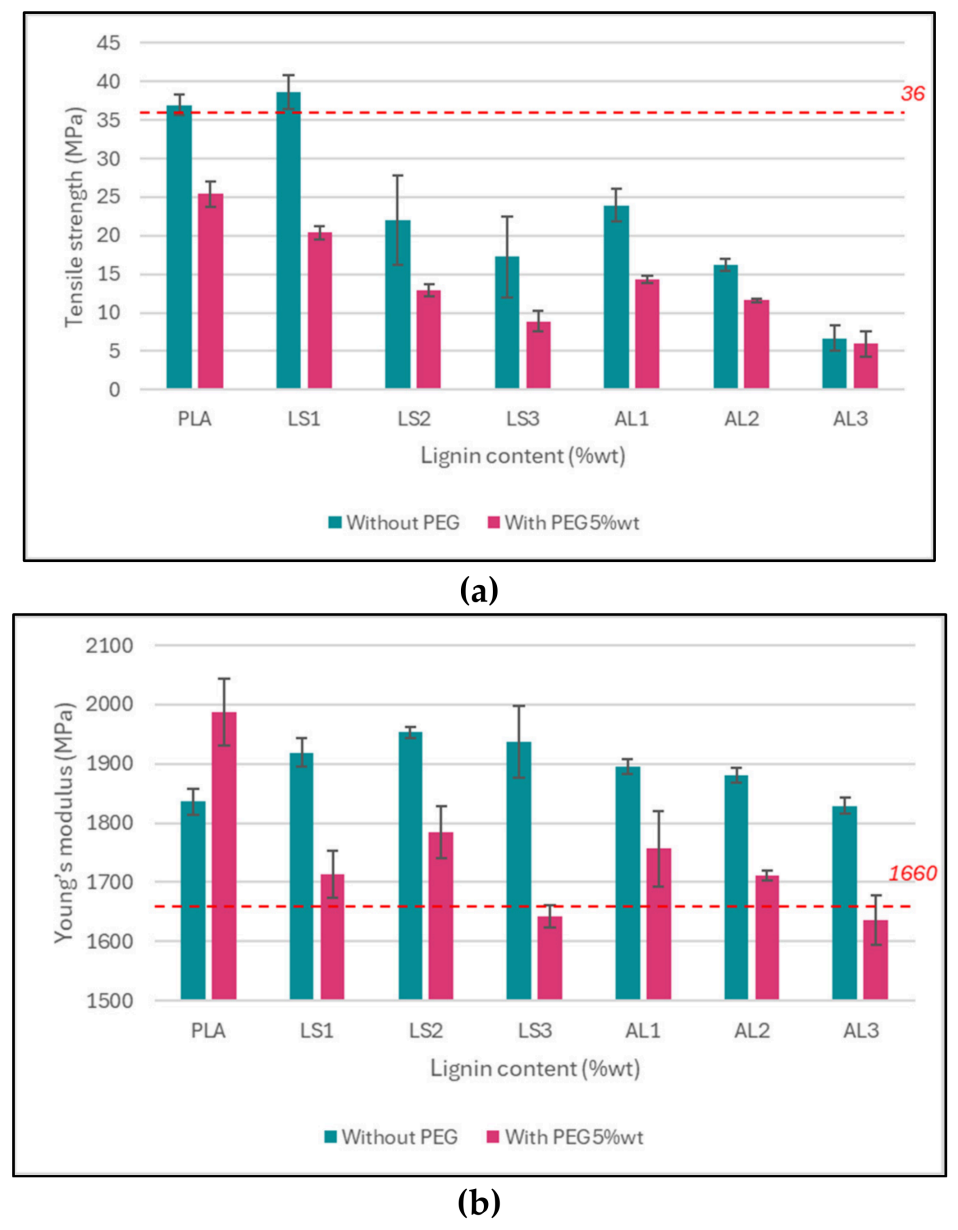
(**a**) Tensile strength and (**b**) Young’s modulus of neat PLA and its biocomposites.

**Figure 4:**
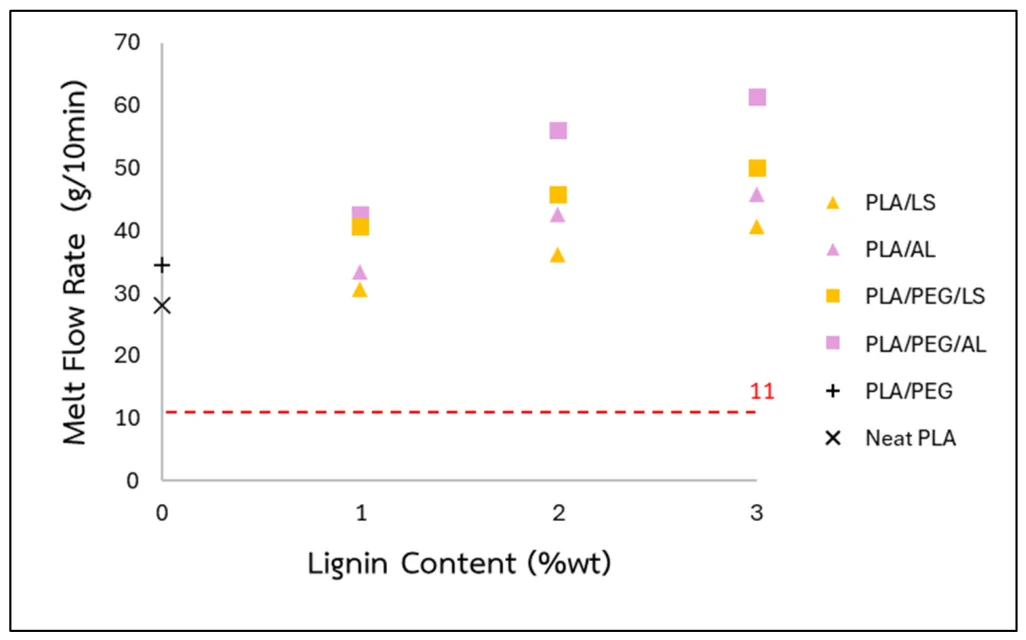
Melt Flow Rate (MFR) of neat PLA and its biocomposites.

**Figure 5:**
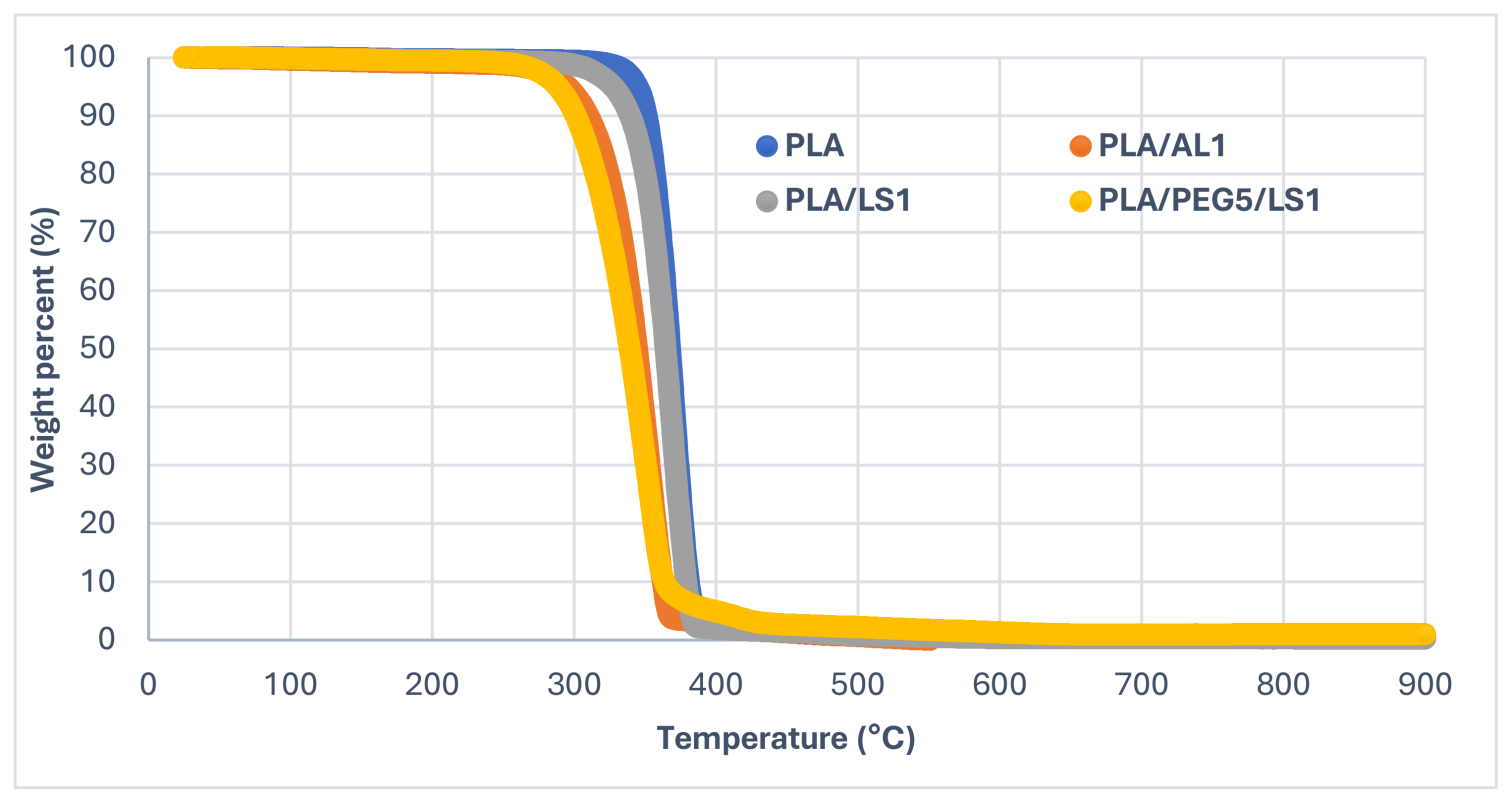
Evolution of weight percent of different composites.

**Figure 6:**
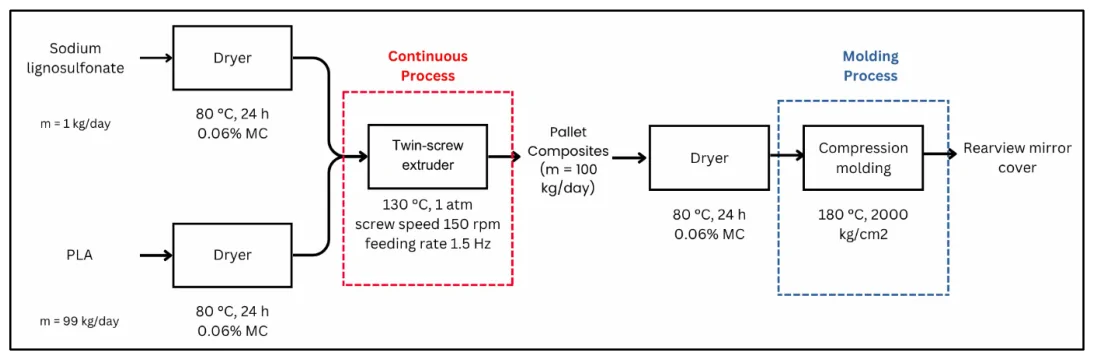
Mass Balance summary-Polylactic acid with 1% sodium lignosulfonate composite for rearview mirror cover.

**Table 1:** Composition of the prepared PLA/lignin/PEG-400 composites.

Lignin Type	PLA Content (%wt.)	Lignin Content (%wt.)	PEG Content (%wt.)
Neat PLA	100	0	0
PLA/PEG	100	0	5
Alkaline lignin	99	1	0
	98	2	0
	97	3	0
	99	1	5
	98	2	5
	97	3	5
Sodium lignosulfonate	99	1	0
	98	2	0
	97	3	0
	99	1	5
	98	2	5
	97	3	5

**Table 2:** Mechanical properties of biocomposites: tensile stress, strain and elongation.

Sample	Tensile Stress (MPa)	Tensile Strain (mm/mm)	Elongation at Break (%)
PLA	36.960	0.028	2.8
PLA/LS1	38.597	0.024	2.4
PLA/LS2	22.023	0.013	1.3
PLA/LS3	17.230	0.010	1.0
PLA/AL1	23.963	0.014	1.4
PLA/AL2	16.200	0.009	0.9
PLA/AL3	6.687	0.004	0.4
PLA/PEG5	23.499	0.013	1.3
PLA/PEG5/LS1	20.399	0.013	1.3
PLA/PEG5/LS2	12.881	0.008	0.8
PLA/PEG5/LS3	8.915	0.009	0.9
PLA/PEG5/AL1	14.343	0.010	1.0
PLA/PEG5/AL2	11.633	0.009	0.9
PLA/PEG5/AL3	5.938	0.006	0.6

**Table 3:** Thermal degradation parameters from TGA analysis.

Composite	T_onset_ (°C)	T_max_ (°C)	Char Residue at 600 °C (%)
Neat PLA	342.2	401.3	1.2
PLA/LS1	327.5	391.0	13.5
PLA/AL1	295.2	374.8	9.1
PLA/PEG5/LS1	288.5	371.8	11.8

**Table 4:** Summary of key economic indicators.

Metric	Value
Production Scale	50,000 units/year
Production Cost/Unit	$0.72
Internal Rate of Return (IRR)	28.5%
Net Present Value (NPV)	$18,160
Payback Period	<4 years

## Data Availability

The data presented in this study are contained within the article. Further inquiries are available on request from the corresponding author.

## Abbreviations

**List of Abbreviations**	
Abbreviation	Full Form
AL	Alkaline Lignin
ASTM	American Society for Testing and Materials
CAGR	Compound Annual Growth Rate
DSC	Differential Scanning Calorimetry
EV	Electric Vehicle
HDT	Heat Deflection Temperature
IRR	Internal Rate of Return
ISO	International Organization for Standardization
LCA	Life Cycle Assessment
LS	Sodium Lignosulfonate
MFR	Melt Flow Rate
NPV	Net Present Value
PEG	Polyethylene Glycol
PLA	Polylactic Acid
SDG	Sustainable Development Goal
SEM	Scanning Electron Microscopy
TEA	Techno-Economic Analysis
TGA	Thermogravimetric Analysis
Tg	Glass Transition Temperature
UTM	Universal Testing Machine
wt.%	Weight Percentage
